# Flavonoids, cytotoxic, antioxidant and antibacterial activities of *Evax pygmaea*

**DOI:** 10.1080/13880209.2017.1405997

**Published:** 2017-11-30

**Authors:** Assia Khalfallah, Djemaa Berrehal, Chawki Bensouici, Ahmed Kabouche, Zahia Semra, Laurence Voutquenne-Nazabadioko, Abdulmagid Alabdul Magid, Zahia Kabouche

**Affiliations:** a Université des frères Mentouri-Constantine, Département de Chimie, Laboratoire d'Obtention de Substances Thérapeutiques (L.O.S.T.), Constantine, Algeria;; b Centre de Recherche en Biotechnologie, Constantine, Algeria;; c CHUC-Benbadis, Bacteriology Service, Constantine, Algeria;; d Groupe Isolement et Structure, Institut de Chimie Moléculaire de Reims (ICMR), CNRS UMR 7312, Reims, France

**Keywords:** DPPH, CUPRAC, metal chelating, β-carotene/linoleic acid, ABTS, MIC

## Abstract

**Context:** Phytochemical study and biological potential of *Evax pygmaea* (L.) Brot. (Asteraceae) are reported for the first time.

**Objective:** To identify the secondary metabolites of *Evax pygmaea* and to determine its antioxidant, antibacterial and cytotoxic activities.

**Materials and methods: **Dried aerial parts (1 kg) were macerated in 70% MeOH (5 L) during 72 h. The concentrated hydromethanolic extract was subjected to extractions with chloroform (3 × 300 mL), ethyl acetate (3 × 300 mL) and *n*-butanol (3 × 300 mL), successively. VLC of combined ethyl acetate (EAEP) and *n*-butanol (BEP) fractions was followed by column purifications. Antioxidant activity was investigated using DPPH, CUPRAC, and metal chelating, β-carotene/linoleic acid and ABTS assays. Agar method was used in the antibacterial study. Cytotoxic activity was determined by Brine shrimp lethality test in DMSO and ethanol, at varying concentrations (2, 1 and 0.2%) and (1, 0.2 and 0.1%) successively.

**Results:** Quercetin (**1**), isorhamnetin 3-*O*-β-d-xyloside (**2**), isorhamnetin 3-*O*-β-d-glucoside (**3**), quercetin 3-*O*-β-d-glucoside (**4**), quercetin 7-*O*-β-D-glucoside (**5**), patuletin 3-*O*-β-d-glucoside (**6**) were isolated from for the first time from *Evax* genus. The EAEP was the most active in ABTS (IC_50_: <3.125 μg/mL) assay whereas the BEEP exhibited the highest activity in the β-carotene/linoleic acid assay (IC_50_: <3.125 μg/mL). The EAEP and BEP exhibited good antibacterial activity (MIC: 40–80 µg/mL). The plant did not show any toxicity (LD_50_>80 µg/mL).

**Discussion and conclusions:** Six flavonoids were isolated for the first time from *Evax pygmaea* which exhibited good antioxidant and antibacterial activities.

## Introduction

Medicinal plants have been widely used for therapeutic purposes since ancient times. The beneficial effects of fruits and vegetables are generally attributed to the presence of phenolic compounds such as phenolic acids, flavonoids and tannins, nitrogen compounds such as alkaloids and amines, as well as vitamins, terpenoids and other metabolites, which have a high antioxidant activity (Cai et al. 2004; Djeridane et al. 2006; Yang et al. 2009).

Asteraceae (Compositae) is the richest vascular plant family in the world, with 1600–1700 genera and 24,000–30,000 species (Funk et al. 2005), it is among the principal families whose species accumulate secondary metabolites with a vast array of important biological activities. Flavonoids are one of these metabolites. Numerous studies have been conducted to prove flavonoids efficacy as antimycotic, antibacterial, antiviral, antiinflammatory, antioxidant, immune modulator, enzyme inhibitor, mutagenic and toxic agents (Havsteen 2002), with antimutagenic and anticancer properties (Harborne 1998).

The genus *Evax* (syn. *Filago*) (Asteraceae) is mainly distributed in the Mediterranean area, it is represented by eight species, five of them are found in Algeria (Quezel and Santa 1963). This genus is not well documented. One paper reported the essential oil composition of *E. pygmaea* (L.) Brot. (syn. *Filago pygmaea* L.), growing wild in Tunisia (Boussaada et al. 2008a) which was characterized by sesquiterpenes (61.9%), diterpenes (33.5%) and monoterpenes (3.4). α-Calacorene (12.2%), cadina-1,4-diene (4.4%), elemol (8.8%), α-cadinol (7.4%), cedrol (5.6%), T-cadinol (4.3%), T-muurolol (4.9%), α-muurolol (3.5%) were the most important components. The second paper described the antimicrobial and antioxidant activities by DPPH and ABTS assays of methanol extracts of the same species collected from Tunisia (Boussaada et al. 2008b).

We report here, for the first time, the phytochemical study, the antioxidant activity, by the use of five methods, and the antibacterial and cytotoxic activities of *E. pygmaea* growing at Constantine (Eastern Algerian) which is known as an antimicrobial.

## Materials and methods

### Chemicals and drugs

NMR spectra were recorded on a Bruker AVANCE DRX 500 NMR spectrometer (Billerica, MA). Thin layer chromatography (TLC) was carried out on precoated silica gel 60 F_254_ (Merck, Kenilworth, NJ), column chromatography (CC) was performed on a polyamide SC6 and silica gel 60 spots were observed under UV light at 254 and 365 nm or visualized by heating after spraying with H_2_SO_4_ 50%. Polyamide SC6 was purchased from Fluka (Buchs, Switzerland).

Both antioxidant and cytotoxic activity measurements were carried out on a 96-well microplate reader, butylatedhydroxylanisole (BHA), butylatedhydroxyltoluene (BHT), 1,1-diphenyl-2-picrylhydrazyl (DPPH^•^), ascorbic acid, 2,2′-azinobis(3-ethyl-benzothiazoline-6-suphonic acid) diammonium salt (ABTS^•+^), β-carotene-linoleic acid were obtained from Sigma Chemical Co. (Sigma-Aldrich GmbH, Steinheim, Germany). All other chemicals and solvents used were of analytical grade.

### Plant material

The aerial parts of *E. pygmaea* were collected on May 2014 at Constantine (North Eastern Algerian) and identified by Professor Gérard De Bélair, Faculty of Sciences University of Annaba, Algeria. A voucher specimen (LOST.Ep.05.14) was deposited at the Herbarium of the Laboratory of Therapeutic Substances (LOST), University des frères Mentouri-Constantine.

### Extraction and isolation of compounds

Air-dried and powdered aerial parts (1.5 kg) of *Evax pygmaea*, were macerated at room temperature with MeOH–H_2_O (70:30, v/v) for 24 h, three times. After filtration and concentration, the residue was dissolved in water. The resulting solution was extracted successively with CHCl_3_, EtOAc and *n*-butanol. Concentration *in vacuo* led to the following fractions: CHCl_3_ (2 g), EtOAc (4.7 g) and *n*-butanol (10 g). Ethyl acetate (EAEP) and *n*-butanol (BEP) fractions were combined because they are similar. This mixture (10 g) was column chromatographed on polyamide SC6 with a gradient of toluene–MeOH with increasing polarity; six main fractions (F3, F5, F7, F8, F10, F11) were collected. Fraction F5 (154 mg), obtained from toluene 88% was further purified on a silica gel CC eluted with an isocratic system (EtOAc:MeOH:H_2_O, 20:0.25:0.25) and yielded a yellow precipitate which was identified as compound **2** (15 mg), the fractions F3 (100 mg), F7 (400 mg), F8 (289 mg), F10 (230 mg) and F11 (320 mg), obtained from toluene 92, 85, 80, 75 and 70%, respectively, yielded the compounds **1**, **3**, **4**, **5** and **6** successively as yellow precipitates (20, 15, 10, 30 and 10 mg, respectively).

The structures of these compounds were established by chemical and spectral analysis UV, NMR and acid hydrolysis as well as by comparing their spectroscopic data with those reported in the literature.

### Acid hydrolysis

The pure compounds were treated with 4 M HCl at 100 °C for 1 h. The hydrolysates were extracted with EtOAc and the aglycones were identified by their UV spectra in methanol and by comparison of their R*f* with authentic samples. Sugars were identified in the aqueous residue by comparison with authentic samples on silica gel TLC impregnated with 0.2 M NaH_2_PO_4_, solvent Me_2_CO–H_2_O (9:1), revealed with aniline malonate. The optical rotation of each purified sugar was measured and compared with authentic samples.

## Antioxidant activity

### DPPH free radical scavenging assay

The free radical-scavenging activity was determined spectrophotometrically by the DPPH assay (Blois 1958; Lakhal et al. 2011). In its radical form, DPPH absorbs at 517 nm, but upon reduction by an antioxidant or a radical species its absorbance decreases. Briefly, a 0.1 mM solution of DPPH^•^ in methanol was prepared and 4 mL of this solution was added to 1 mL of sample solutions in methanol at different concentrations. Thirty minutes later, the absorbance was measured at 517 nm. Lower absorbance of the reaction mixture indicated higher free radical-scavenging activity. BHT and BHA were used as antioxidant standards for comparison of the activity. The scavenging capability of DPPH radical was calculated using the following equation. The results were given as IC_50_ (µg/mL) corresponding the concentration of 50% inhibition.

$$\text{DPPH scavenging effect }(\text{\%})=\frac{A_{\text{Control}}-A_{\text{Sample}}}{A_{\text{Control}}}\times 100$$

where *A*_control_ and *A*_sample_ are the absorbances of the reference and sample obtained from the UV–visible spectrophotometer, respectively.

### Metal chelating activity assay

The metal chelating activity by the ferrene–Fe^2+^ complexation assay measured spectrophotometrically (Decker and Welch 1990; Labed et al. 2016) with slight modifications. The extracts solution (80 μL dissolved in ethanol in different concentrations) were added to 40 μL 0.2 mM FeCl_2_. The reaction was initiated after addition of 80 μL 0.5 mM ferene. The mixture was shaken then left at room temperature for 10 min. At the equilibrium, the absorbance was measured at 593 nm. The activity was calculated by the use of the following equation. The results were given as IC_50_ value (mg/mL) (50% inhibition):

$$\text{Metal chelating activity }(\text{\%})=\frac{A_{\text{Control}}-A_{\text{Sample}}}{A_{\text{Control}}}\times 100$$

### ABTS cation radical decolourization assay

The ABTS^•+^ scavenging activity was determined according to the method of Re et al. (1999). The ABTS^•+^ was generated by the reaction between 7 mM ABTS in water and 2.45 mM potassium persulfate, stored in the dark at room temperature for 12 h. The ABTS^•+^ solution was diluted to get an absorbance of 0.703 ± 0.025 at 734 nm with ethanol which was used as a control. BHT and BHA were used as antioxidant standards and the activity was calculated by the use of the following equation. The results were given as IC_50_ (µg/mL)

$${\text{ABTS}}^{•+}\text{ scavenging activity }(\text{\%})=\frac{A_{\text{Control}}-A_{\text{Sample}}}{A_{\text{Control}}}\times 100$$

### β-Carotene/linoleic acid assay

The bleaching activity of the fractions was evaluated by the β-carotene-linoleic acid test system (Miller 1971; Ferhat et al. 2017). β-Carotene (0.5 mg) in 1 mL of chloroform and 25 μL of linoleic acid were dissolved in 200 mg of Tween 40 emulsifier mixture. After evaporation of chloroform under vacuum, 100 mL of distilled water saturated with oxygen, were added by vigorous shaking. Four millilitres of this mixture were transferred into different test tubes containing different concentrations of the sample. As soon as the emulsion was added to each tube, the zero time absorbance was measured at 470 nm using spectrophotometer. The emulsion system was incubated for 2 h at 50 °C. Ethanol was used as a control whereas BHA and BHT were used as antioxidant standards. The results were given as 50% inhibition concentration (IC_50_).

The bleaching rate (*R*) of β-carotene was calculated according to the following equation:

$$R=\frac{\ln\;a/b}{t}$$

where ln is the natural log, *a* is the absorbance at time zero and *b* is the absorbance at time *t* (120 min). The antioxidant activity was calculated in terms of percent inhibition relative to the control, using following equation:

$$\text{Antioxidant activity }(\text{\%})=\frac{R_{\text{Control}}-R_{\text{Sample}}}{R_{\text{Control}}}\times 100$$

### Cupric reducing antioxidant capacity (CUPRAC) assay

The cupric reducing capacity of the fractions was determined by the CUPRAC method (Apak et al. 2004; Bensouici et al. 2016). One millilitres of copper (II) chloride solution (0.01 M prepared from CuCl_2_·2H_2_O), 1 mL of ammonium acetate buffer at pH 7.0 and 1 mL of neocaproin solution (0.0075 M) were mixed to 0.5 mL of plant extract or standard of different concentrations solution. The final volume of the mixture was adjusted to 4.1 mL by adding 0.6 mL of distilled water. The resulting mixture was incubated for 1 h at room temperature, and then the absorbance of the solution was measured at 450 nm by the use of a spectrophotometer against blank and BHT and BHA as standards.

The results were given as *A*_0.5_ (µg/mL) corresponding the concentration indicating 0.50 absorbance intensity.

### Polyphenol content

The total polyphenolics was determined by the Folin–Ciocalteu method, with slight modifications. Mixture of extract (0.5 mL) and 5 mL of Folin–Ciocalteu reagent (1 mol) was neutralized with 4 mL saturated sodium carbonate (75 g/L), and kept at room temperature for 2 h. The absorbance was measured at 760 nm by the use of a spectrophotometer. The total polyphenolics were expressed as gallic acid equivalents (GAE) (Singleton et al. 1999).

### Determination of the antibacterial activity

Susceptibility of the bacterial strains namely *Escherichia coli* ATCC 25922, *Staphylococcus aureus* ATCC 43300, *Pseudomonas aeruginosa* ATCC 27853, *Klebsiella pneumonia, Salmonella heilberg*, *Shigella sonnei* and *Enterobarter aerogenes* to the BEP, EAEP and CEP fractions of *Evax pygmaea* were investigated using the agar method (NCCLS 2007). The reference strains were obtained from the Pasteur Institute (Algiers). The other strains were clinically obtained from the laboratory of bacteriology, Benbadis Hospital, Constantine, using conventional methods (clinical isolation). Gentamycin was used as reference. The activity was estimated visually by the presence or absence of colonies. MIC values were recorded as the lowest concentrations of compounds showing no growth. This test was performed in triplicate.

## Cytotoxic activity

### Brine shrimp lethality test

Cytotoxicity of the BEP, EAEP and CEP fractions of *E. pygmaea* was evaluated using Brine shrimp lethality assay (Meyer et al. 1982). Each extract (100 μL) was incubated, for 24 h under lighting, with 10 larvae of *Artemia* adjusted until 5 mL of 70% salty sea water (five tubes were prepared for each extract). Control larvae were placed in DMSO and ethanol, at varying concentrations (2, 1 and 0.2%) and (1, 0.2 and 0.1%) successively. After 24 h, the tubes were examined against a lighted background and the average number of larvae that survived in each tube was determined.

Mortality: % Deaths (% deaths test - % control deaths)/(100 - % control deaths)

The concentration at 50% mortality of the larvae (LC_50_) was determined using the microplate reader.

### Statistical analyses

All data on activity tests were the averages of triplicate analyses. The data were recorded as means ± standard error meaning. Significant differences between means were determined by Student’s *t-*test; *p* values <0.05 were regarded as significant.

## Results and discussion

### Identification of compounds 1–6

**Figure 1.**
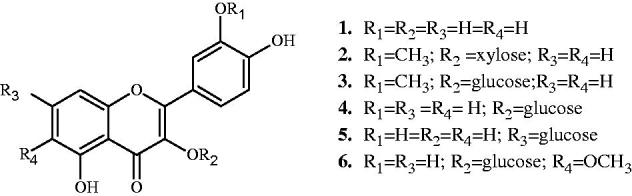
Structures of compounds (**1**–**6**) isolated from *Evax pygmaea*.

Six known flavonoids, quercetin (**1**) (Bencheraiet et al. 2011), isorhamnetin 3-*O*-β-d-xyloside (**2**) (Agrawal and Bansal 1989), isorhamnetin 3-*O*-β-d-glucoside (**3**) (Benmekhbi et al. 2008), quercetin 3-*O*-β-d-glucoside (**4**) (Touafek et al. 2011), quercetin 7-*O*-β-d-glucoside (**5**) (Sikorska & Matławska 2000), patuletin 3-*O*-β-d-glucoside (**6**) (Nacer et al. 2006) (Figure 1), were isolated from the BEP and EAEP fractions of *Evax pygmaea* and identified by the use of UV, NMR and acid hydrolysis.

### Quercetin (1)

C_15_H_10_O_7_, yellow powder, UV (MeOH, *λ_max_*, nm): 257, 375; +NaOH: 271, 425; +AlCl_3_: 270, 452; +AlCl_3_/HCl: 267, 426; NaOAc: 274, 394. +H_3_BO_3_: 269, 365. ^1^H NMR (500 MHz, CD_3_OD, *δ*, ppm, *J*/Hz): 7.74 (1H, *d*, *J* = 2.0, H-2′), 7.65 (1H, *dd*, *J* = 8.5; 2.0, H-6′), 6.92 (1H, *d*, *J* = 8.5, H-5′), 6.41 (1H, *d*, *J* = 2.1, H-8), 6.21 (1H, *d*, *J* = 2.1, H-6).

### Isorhamnetin 3-*O*-β-d-xyloside (2)

C_21_H_20_O_11_, yellow powder, UV (MeOH, *λ_max_*, nm): 253, 345; +NaOH: 271, 335, 415, +AlCl_3_: 268, 401; +AlCl_3_/HCl: 268, 402; NaOAc: 275, 395. +H_3_BO_3_: 270, 346. ^1^H NMR (500 MHz, CD_3_OD, *δ*, ppm, *J*/Hz): 7.86 (1H, *d*, *J* = 2.0, H-2′), 7.57 (1H, *dd*, *J* = 8.4; 2.0, H-6′), 6.93 (1H, *d*, *J* = 8.4, H-5′), 6.44 (1H, *d*, *J* = 2.1, H-8), 6.19 (1H, *d*, *J* = 2.1, H-6), 5.37 (1H, *d*, *J* = 7.25, H-1″ xylose), 2.70–3.70 (sugar protons), 3.84 (3H, *s*, OCH_3_).

### Isorhamnetin 3-*O*-β-d-glucoside (3)

C_22_H_22_O_12_, yellow powder, UV (MeOH, *λ_max_*, nm): 254, 355; +NaOH: 271, 330, 413, +AlCl_3_: 268, 403; +AlCl_3_/HCl: 269, 400; NaOAc: 274, 396. +H_3_BO_3_: 269, 365. ^1^H NMR (500 MHz, CD_3_OD, *δ*, ppm, *J*/Hz): 7.95 (1H, *d*, *J* = 2.0, H-2′), 7.61 (1H, *dd*, *J* = 8.5; 2.0, H-6′), 6.93 (1H, *d*, *J* = 8.5, H-5'), 6.43 (1H, *d*, *J* = 2.1, H-8), 6.23 (1H, *d*, *J* = 2.1, H-6), 5.44 (1H, *d*, *J* = 7.4, H-1″ glucose), 3.20–3.90 (sugar protons), 3.76 (3H, *s*, OCH_3_).

### Quercetin 3-*O*-β-d-glucoside (4)

C_21_H_20_O_12_, yellow powder, UV (MeOH, *λ_max_*, nm): 256, 357; +NaOH: 273, 328, 409, +AlCl_3_: 269, 435; +AlCl_3_/HCl: 269, 403; NaOAc: 273, 392. +H_3_BO_3_: 264, 380. ^1^H NMR (500 MHz, CD_3_OD, *δ*, ppm, *J*/Hz): 7.74 (1H, *d*, *J* = 2.0, H-2′), 7.61 (1H, *dd*, *J* = 8.5; 2.0, H-6′), 6.92 (1H, *d*, *J* = 8.5, H-5′), 6.41 (1H, *d*, *J* = 1.8, H-8), 6.23 (1H, *d*, *J* = 1.8, H-6), 5.27 (1H, *d*, *J* = 7.6, H-1″ glucose), 3.20–3.79 (sugar protons).

### Quercetin 7-*O*-β-d-glucoside (5)

C_21_H_20_O_12_, yellow powder, UV (MeOH, *λ_max_*, nm): 256, 372; +NaOH: 271, 425; +AlCl_3_: 271, 461; +AlCl_3_/HCl: 267, 429; NaOAc: 259, 406. +H_3_BO_3_: 261, 390. ^1^H NMR (500 MHz, CD_3_OD, *δ*, ppm, *J*/Hz): 7.79 (1H, *d*, *J* = 2.1, H-2′), 7.69 (1H, *dd*, *J* = 8.4; 2.1, H-6′), 6.91 (1H, *d*, *J* = 8.4, H-5′), 6.78 (1H, *d*, *J* = 2.1, H-8), 6.49 (1H, *d*, *J* = 2.1, H-6), 5.08 (1H, *d*, *J* = 7.4, H-1″ glucose), 3.40–3.75 (sugar protons).

### Patuletin 3-*O*-β-d-glucoside (6)

C_22_H_22_O_13_, yellow powder, UV (MeOH, *λ_max_*, nm): 263, 360; +NaOH: 269, 408; +AlCl_3_: 276, 456; +AlCl_3_/HCl: 273, 395; NaOAc: 261, 366. +H_3_BO_3_: 271, 381. ^1^H NMR (500 MHz, CD_3_OD, *δ*, ppm, *J*/Hz): 7.80 (1H, *d*, *J* = 2.1, H-2′), 7.69 (1H, *dd*, *J* = 8.5; 2.1, H-6′), 6.90 (1H, *d*, *J* = 8.5, H-5′), 6.69 (1H, *s,* H-8), 5.08 (1H, *d*, *J* = 7.2, H-1″ glucose), 3.10-4.10 (sugar protons), 3.71 (3H, *s*, OCH_3_).

### Antioxidant properties

The BEP, EAEP and CEP fractions of *Evax pygmaea* were evaluated for their antioxidant activity. The results showed that the EAEP was the most active in DPPH (IC_50_: 6.91 ± 0.09 µg/mL), ABTS (IC_50_: <3.125 μg/mL) and CUPRAC (*A*_0.50_: 6.26 ± 0.04 μg/mL); this can be due to its polyphenol content which is higher than that of BEP (Table 1). The results obtained with the DPPH assay may be explained by the flavonoid content with isorhamnetin and quercetin major squeletons (Figure 1). In their study of the structure–activity relationships, Muhammad et al. (2016) showed that the position of –OH and –OCH_3_ has a great influence on the antioxidant activity.

**Table 1.** Total polyphenolic content of ethyl acetate (EAEP) and *n*-butanol (BEP) fractions of *Evax pygmaea*^a^.

Plant fractions	Total phenolic content (mg/g)^b^
EAEP	349.69 ± 2.20
BEP	273.31 ± 2.60

a Values are mean ± SD (*n* = 3).

b mg/g gallic acid equivalent.

In the metal chelating inhibition, the CEP was the highest (>50% at 100 µg/mL), however, the BEP exhibited the best activity in the β-carotene/linoleic acid decolourization assay (IC_50_: <3.125 μg/mL) (Table 2).

**Table 2.** Antioxidant activity of *E. pygmaea* fractions by the DPPH, ABTS, CUPRAC, metal chelating and β-carotene assays^a^.

	Antioxidant activity				
	DPPH assay	ABTS assay	β-Carotene assay	CUPRAC assay	Metal chelating assay
Extracts	IC_50_ (µg/mL)			*A*_0.50_ (µg/mL)	% Inhibition at 100 µg/mL
CEP	49.21 ± 0.26	10.61 ± 0.38	9.01 ± 1.19	31.16 ± 1.02	>50
EAEP	6.91 ± 0.09	<3.125	4.07 ± 1.12	6.26 ± 0.04	3.74 ± 0.61
BEP	10.47 ± 0.18	8.88 ± 0.19	<3.125	11.66 ± 0.76	13.95 ± 1.00
BHA^b^	5.73 ± 0.41	1.81 ± 0.10	0.90 ± 0.02	3.64 ± 0.19	8.41 ± 0.67
BHT^b^	22.32 ± 1.19	1.29 ± 0.30	1.05 ± 0.01	9.62 ± 0.87	>50
Ascorbic acid	–	–	–	–	9.01 ± 1.46

a IC_50_ and A_0.50_ values expressed are means ± SD of three parallel measurements (*p* < 0.05).

b Reference compounds: BHA: butylated hydroxyanisole; BHT: butylated hydroxytoluene.

### Antibacterial activity

The EAEP and BEP fractions displayed the highest activity against *Escherichia coli* ATCC 25922, *Staphylococcus aureus* ATCC 43300 and *Pseudomonas aeruginosa* ATCC 27853, with 40 µg/mL MIC’s value (Table 3). However, the CEP fraction inhibited the growth of almost tested microorganisms with 80 µg/mL MIC’s value except against *Klebsiella pneumoniae* (MIC 40 µg/mL).

**Table 3.** Antibacterial activity (MICs) of the chloroform (CEP), ethyl acetate (EAEP) and *n*-butanol (BEP) fractions of *Evax pygmaea*.

Bacteria	MIC^a^ (µg/mL)			
	CEP	EAEP	BEP	Genta^b^
*Escherichia coli* ATCC 25922^c^	80 ± 0.50	40 ± 0.20	40 ± 0.70	10 ± 0.20
*Staphylococcus aureus* ATCC 43300^c^	80 ± 1.20	40 ± 1.00	40 ± 2.30	15 ± 0.30
*Pseudomonas aeruginosa* ATCC 27853^c^	80 ± 1.50	40 ± 0.80	40 ± 1.90	5 ± 0.10
*Klebsiella pneumoniae*^d^	40 ± 0.50	80 ± 0.20	–	5 ± 0.20
*Salmonella heilberg*^d^	80 ± 2.50	80 ± 0.80	80 ± 1.70	–
*Shigella sonnei*^d^	80 ± 0.35	–	–	–
*Enterobarter aerogenes*^d^	80 ± 1.90	80 ± 1.40	80 ± 0.70	–

a Values are mean ± SD (*n* = 3).

b Gentamycin: 10 µg/mL.

c Obtained from the Pasteur Institute (Algiers).

d Clinical isolates from the laboratory of bacteriology (CHU Constantine, Algeria).

### Cytotoxic activity

Cytotoxicity analysis, determined by Brine shrimp lethality test, revealed that this plant was not toxic as their LD_50_ values were all greater than 80 μg/mL (Table 4).

**Table 4.** Cytotoxic activity of extracts of *E. pygmaea*.

	% Mortality rate^a^					
Plant fractions	10 µg	20 µg	40 µg	80 µg	LD_50_^b^ µg/mL	Toxicity
EAEP	0.00 ± 0.00	0.00 ± 0.00	0.00 ± 0.00	3.33 ± 1.77	>80	–
BEP	0.00 ± 0.00	0.00 ± 0.00	6.67 ± 1.55	13.33 ± 1.77	>80	–
CEP	3.33 ± 1.77	6.67 ± 1.55	13.33 ± 5.77	20.00 ± 00.00	>80	–

a Values expressed are means ± SD of three parallel measurements (*p*< 0.05).

b LD_50_: median lethal dose.

## Conclusions

Six flavonoids have been isolated, for the first time, from the ethyl acetate and *n*-butanol fractions of *E. pygmaea.* These fractions showed a good antioxidant activity with the five used methods namely, β-carotene-linoleic acid, DPPH^•^ and ABTS^•+^ scavenging, CUPRAC and metal chelating assays. This result could be in part related to the isolated flavonoids from which some possess a quercetin squeleton. The fractions showed a good antibacterial activity against tested microorganisms which confirms its use as an antimicrobial. The brine shrimp lethality test revealed that this plant was not toxic. The results of this study indicated that *E. pygmaea* could be an important dietary source of phenolic compounds with high antioxidant capacity.
